# Three to Tango: MUC1 as a Ligand for Both E-Selectin and ICAM-1 in the Breast Cancer Metastatic Cascade

**DOI:** 10.3389/fonc.2012.00076

**Published:** 2012-07-27

**Authors:** Yue Geng, Kimberly Yeh, Tait Takatani, Michael R. King

**Affiliations:** ^1^Department of Biomedical Engineering, Cornell UniversityIthaca, NY, USA

**Keywords:** adhesion, breast cancer, circulation, E-selectin, ICAM-1, MUC1

## Abstract

Cancer cell tethering and rolling on the vascular wall is facilitated by various selectin: glycoprotein interactions which lead to eventual extravasation and metastases. The aberrantly underglycosylated mucin MUC1 has been shown to both abundantly express selectin binding moieties (sialyl Lewis x and a) and to consistently expose its core epitope. Flow cytometry was used to determine MUC1 expression on ZR-75-1 and MCF7 cells, while immunofluorescence microscopy was used to confirm the aberrant form of MUC1 and MUC1:ICAM-1 interactions. Each cell line was then perfused through combined E-selectin and ICAM-1 coated microtubes, as a model of the microvascular endothelium. ZR-75-1 and MCF7 were found to express abundant and low levels of underglycosylated MUC1, respectively. The rolling/adhesion profiles showed that ZR-75-1 cells, when compared to MCF7 cells, interact with E-selectin more efficiently resulting in sufficiently slow rolling velocities to form MUC1:ICAM-1 interactions thereby facilitating firm adhesion. The purpose and novelty of this work is the demonstration of the synergistic adhesion capabilities of MUC1 in the metastatic adhesion cascade, where the observed differential adhesion is consistent with the relative metastatic potential of the ZR-75-1 (highly metastatic) and MCF7 (weakly metastatic) cell lines.

## Introduction

The mucin family of glycoproteins is traditionally associated with the protection of the epithelial layer and provides lubrication of luminal epithelial surfaces. More recently, certain mucins have been identified as markers for metastatic cancers. Of particular importance, the mucin MUC1 is overexpressed in numerous cancers including breast, ovarian, lung, pancreatic, prostate, gastric, and colorectal (Zotter et al., 1987; Girling et al., 1989; Ajioka et al., 1996; Burdick et al., 1997; Retz et al., 1998), where high expression generally correlates with increased mortality rates (MacLean et al., 1997; Guddo et al., 1998; Kocer et al., 2004; Duncan et al., 2007; Tewes et al., 2009). It is then reasonable to hypothesize that cancer’s adaptation and alteration of MUC1 may play a vital role in metastatic progression.

Cancer metastasis through the bloodstream is initiated by the invasion of tumor cells from the primary site into the blood vessel (Moss and Anderson, 2000). These circulating tumor cells (CTCs) can then adhere to the endothelial lining, which leads to extravasation and the formation of secondary tumor sites. For CTC adhesion, cells may first establish transient interactions with the activated endothelium which facilitates cell tethering and rolling events (Tremblay et al., 2008; St Hill, 2011; Wirtz et al., 2011). These types of interactions are produced via selectins, a family of adhesion molecules expressed by the endothelium, and carbohydrate moieties, such as sialyl Lewis x (sLe^x^) or sialyl Lewis a (sLe^a^), present on the selectin ligands expressed by CTCs (Borsig et al., 2002; Varki and Varki, 2007). Once the cell has sufficiently reduced its rolling velocity, firm adhesion can be acquired through the interaction between the intracellular cell adhesion molecule 1 (ICAM-1) on the endothelium and integrins on CTCs. This series of events is commonly referred to as the metastatic adhesion cascade (Orr et al., 2000).

MUC1 is not only overexpressed in many cancer types but is also aberrantly underglycosylated. The core structure of the extracellular domain of MUC1 mainly consists of 25–150 repeat units of 20 identical amino acid sequences rich in serines and threonines resulting in a length 5–10 times that of most membrane proteins. Normally, these amino acids would be richly O-glycosylated however aberrant MUC1 has been shown to express shortened oligosaccharides such as sLe^x^ and sLe^a^, and binds efficiently to E-selectin (Lloyd et al., 1996). Interestingly, high levels of MUC1 carrying sLe^x/a^ correlate to poor prognosis in patients with lung adenocarcinoma (Inata et al., 2007). Aberrant MUC1 also has the propensity to expose its core epitope due to underglycosylation where it has become the target of various probes to determine MUC1 expression (Moore et al., 2004). Furthermore, ICAM-1 has been shown to recognize and bind to the core epitope of MUC1 (Hayashi et al., 2001). Therefore, CTCs may utilize aberrant MUC1 to facilitate tethering and rolling due to the increased length of MUC1 relative to other selectin ligands, and firmly adhere to the endothelium via ICAM-1 interactions (Regimbald et al., 1996).

In this study, we investigate the role of MUC1 in breast cancer cell adhesion under flow with two cell lines: ZR-75-1 which is known to have a high metastatic potential, and MCF7 which is weakly metastatic. The differential adhesion of these two cell lines to the endothelium are studied *in vitro* via micro-renathane tubes coated with varying ratios of E-selectin and ICAM-1 which represent a model of metastasis-prone microvasculature (Finzel et al., 2004). We hypothesize that the underglycosylated form of MUC1 may serve as a ligand for both E-selectin and ICAM-1, which would allow for efficient interaction between CTCs in transit and the inflamed endothelium.

## Materials and Methods

### Reagents

Recombinant E-selectin-IgG_1_ chimera and recombinant ICAM-1-IgG_1_ chimera were purchased from R&D systems (Minneapolis, MN, USA). Blotting grade blocker non-fat dry milk was obtained from Bio-Rad Laboratories (Hercules, CA, USA) and Protein-G was purchased from EMD Biosciences (San Diego, CA, USA). FITC mouse anti-human CD227 (clone HMPV), FITC mouse IgG1 k isotype control, purified mouse anti-human CD15s (clone CSLEX), APC rat anti-mouse IgM, and FITC goat anti-mouse IgG/IgM were all purchased from BD Biosciences (San Jose, CA, USA). FITC mouse anti-human CD44v4 was obtained from AbD Serote (Germany). FITC and APC anti-human IgG antibodies were purchased from Invitrogen (Carmarillo, CA, USA). Ca^2+^ and Mg^2+^ free DPBS (Invitrogen, Camarillo, CA, USA), calcium carbonate (Sigma Chemical Co., St. Louis, MO, USA), low endotoxin (1 ng/mg), and essentially globulin-free Bovine Serum Albumin (Sigma Chemical Co., St. Louis, MO, USA) were used to prepare flow buffer for cell adhesion assays.

### Breast cancer cell culture

Breast cancer cell lines ZR-75-1 and MCF7 were purchased from ATCC and maintained in 10% Fetal Bovine Serum (FBS; Cellgro), 1% penicillin-streptomycin (Invitrogen), and RPMI 1640 medium (ZR-75-1) or eagle’s minimal essential medium with 0.01 mg/mL bovine insulin (MCF7) at 37°C with 5% CO_2_ in a humidified incubator.

### Flow cytometry

Cells were removed from tissue culture flasks prior to antibody incubation using an enzyme-free cell dissociation buffer solution. After washing with 1× DPBS, the cells were resuspended in 1% BSA in DPBS to a final concentration of approximately 250,000 cells in each sample. Antibodies against MUC1 or appropriate isotype controls were added to the cell suspensions and incubated over ice for 45 min. Specifically, mouse anti-human MUC1 mAb clone HMPV (reacts with the core peptide of MUC1) and mouse anti-human MUC1 mAb clone SM3 (recognizes the underglycosylated form of MUC1) were used in this study. Following incubation, cells were washed twice with 500 μL of 1% BSA to remove any unbound antibody. Flow cytometry samples were analyzed using a BD Accuri C6 flow cytometer (Ann Arbor, MI, USA).

### Soluble ICAM-1 binding assay

Recombinant human ICAM-1-IgG_1_ chimeric protein (R&D) was fluorescently tagged with Alexa 647 anti-human IgG antibody and incubated with ZR-75-1 and MCF7 cells in 1× DPBS with 2% BSA for 30 min at room temperature. Unbound proteins were washed off with 1× DPBS twice prior to flow cytometry and confocal microscopy imaging.

### Preparation of combined protein surfaces

Micro-renathane tubings (microtubes) with an inner diameter of 300 μm (Braintree Scientific Inc., Braintree, MA, USA) were cut to lengths of 50 cm. Recombinant human E-selectin-IgG_1_ and ICAM-1-IgG_1_ chimeric proteins were each dissolved in 1× PBS and mixed in various ratios (E-sel/ICAM-1: 10/0, 7.5/2.5, 5/5, 2.5/7.5, 0/10) to a final protein concentration of 10 μg/mL. The microtube surface was first rinsed with 1× DPBS and then incubated with 10 μg/mL of protein-G solution for 1 h, followed by a 2 h incubation with the premixed E-selectin and ICAM-1 protein solution, then blocked with 5% milk protein in PBS for 1 h. To evaluate the correlation between incubation concentrations and surface coverage, FITC conjugated E-selectin and APC conjugated ICAM-1 were mixed in the ratios described above. Fluorescence images were taken and analyzed using Image J.^1^

### Cell adhesion assay

After surface functionalization as described above, microtubes were secured to the stage of an Olympus IX81 motorized inverted microscope (Olympus America, Melville, NY, USA). A CCD camera (model no: KP-M1AN, Hitachi, Tokyo, Japan) and a DVD recorder (model no: DVD-1000MD, Sony Electronics) were used to record experiments for offline analysis. ZR-75-1 and MCF7 breast cancer cells suspended in flow buffer were perfused through protein coated microtubes using a syringe pump (KDS 230, IITC Life Science, Woodland Hills, CA, USA) at a wall shear stress of 1.0 dyne/cm^2^.

### Confocal immunofluorescence microscopy

ZR-75-1 and MCF7 cells were removed from tissue culture flasks, washed with 1× DPBS, resuspended with 2% BSA in 1× DPBS, loaded to a pre-assembled cytospin cuvette, and spun at 750 rpm for 3 min in a Shandon Cytospin 3 centrifuge (Harlow Scientific, Arlington, MA, USA). Cytospin slides were then dried and fixed with 4% paraformaldehyde (Electron Microscopy Sciences, Hatfield, PA, USA) prior to antibody labeling. Indirect surface staining for MUC1 was performed using mouse anti-human MUC1 mAb (clone SM3) and Alexa 647 rat anti-mouse mAb as a secondary antibody. For some cytospin slides, nuclear staining with DAPI was performed for 10 min at room temperature prior to imaging. Samples from the soluble ICAM-1 binding assay were deposited on cytospin slides for imaging. A Zeiss 710 laser scanning confocal microscope at the Cornell University microscopy and imaging core facility was used to collect images using a 40× objective.

### Data acquisition and analysis

“Rolling” cells were defined as those observed to translate in the direction of flow with an average velocity less than 50% of the calculated hydrodynamic free stream velocity. The rolling velocity was calculated by measuring the distance a rolling cell traveled over a 30 s interval. Videos of rolling cells were taken at three randomly selected locations along the microtube. “Tethering” cells were defined as cells that were observed to roll intermittently with fluctuating velocity. The quantity of cells rolling, adherent and tethering was determined by recording images at 30 randomly selected locations along the microtube. All errors are reported as standard error of the mean, and statistical analyses were performed using Prism (GraphPad Software, San Diego, CA, USA).

### Molecular dynamics

The crystal structure of SM3 bound to the MUC1 core fragment (1SM3; Dokurno et al., 1998) was obtained from the Protein Data Bank for use as starting coordinates. The MUC1 fragment was equally extended beyond the SM3:MUC1 interaction to include all amino acids of one complete tandem repeat unit (PATSGPAPRTDPASTVGHAP) and the furthest threonine/serine from SM3 was O-glycosylated with the sLe^x^ carbohydrate group. Using the YASARA^2^ package of molecular dynamics (MD) programs, the complex was solvated in a water cube with an initial length of 100 Å to allow for free protein rotation and neutralized to 0.9% NaCl with physiologically neutral pH (7.4). The YAMBER3 self-parameterizing force field (Krieger et al., 2004) was implemented with periodic boundary conditions, the particle mesh Ewald method for electrostatic interactions (Essmann et al., 1995) and a recommended 7.86 Å force cutoff for long-range interactions. Temperature and pressure were held constant at 300 K and 1 atm, respectively, while the water box was allowed to adjust slightly to constrain the water density to 0.997 g/L. Conformational stresses were then removed via short steepest descent minimizations and simulated annealing was run until sufficient convergences were reached. A free dynamics simulation was then run for 10 ns to obtain the final equilibrated structure.

## Results

### Differential MUC1 expression on ZR-75-1 and MCF7 cells and their ability to bind to ICAM-1 under static conditions

**Figure 1:**
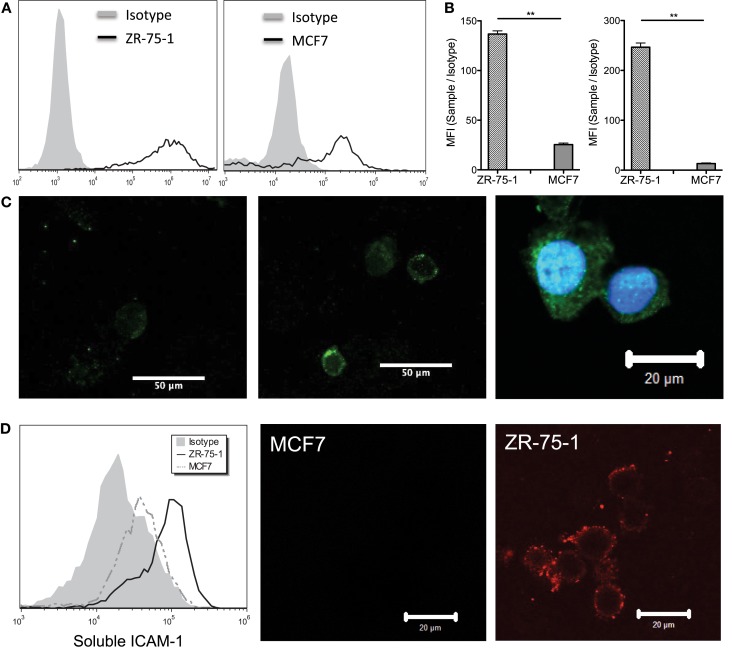
**(A)** Flow cytometry histogram plots of ZR-75-1 and MCF7 labeled with anti-MUC1 mAb clone HMPV, respectively. **(B)** Quantification of the MFI ratio of sample/isotype for both cell types with anti-MUC1 mAb clone SM3 and HMPV, respectively. Student’s *t*-test was used for statistical analysis and results from both labeling experiments were found to be significantly different for ZR-75-1 and MCF7 cells (*p* ≤ 0.01). **(C)** Left and middle: confocal microscopy images of MCF7 and ZR-75-1 labeled with anti-MUC1 mAb (clone SM3), respectively. Right: strong signal of SM3 anti-MUC1 mAb in the cytoplasmic region of select ZR-75-1 cells. **(D)** Left: flow cytometry histogram of fluorescently tagged ICAM-1 labeling on ZR-75-1 and MCF7 cells. Middle and right: confocal microscopy images of MCF7 and ZR-75-1 cells labeled with fluorescently tagged ICAM-1.

MUC1 core peptide expression was measured for both ZR-75-1 and MCF7 cells via flow cytometry using MUC1 mAb clone HMPV and found to be significantly higher on ZR-75-1 cells (Figure 1A). MUC1 mAb clone SM3 was also used to detect the underglycosylated form of MUC1, which has been identified as a tumor associated form of MUC1 (Mommers et al., 1999). Although no significant shift was observed, mean fluorescence intensity (MFI) of sample/isotype for ZR-75-1 cells was observed to be five times higher than MCF7 cells (Figure 1B). Furthermore, confocal microscopy images with MUC1 antibody (SM3) labeling showed brighter signals on ZR-75-1 cells (Figure 1C). Strong homogenous cytoplasmic staining of MUC1 (SM3) was observed on some ZR-75-1 cells (Figure 1C right) but not on MCF7 cells. To assay MUC1:ICAM-1 binding under static conditions, human recombinant ICAM-1 was conjugated with a fluorescently tagged secondary antibody and incubated with both cell types. Flow cytometry and confocal microscopy results both showed significantly stronger binding of ICAM-1 to ZR-75-1 cells compared to MCF7 cells (Figure 1D).

### E-selectin ligand and binding moiety expression on ZR-75-1 and MCF7 cells

**Figure 2:**
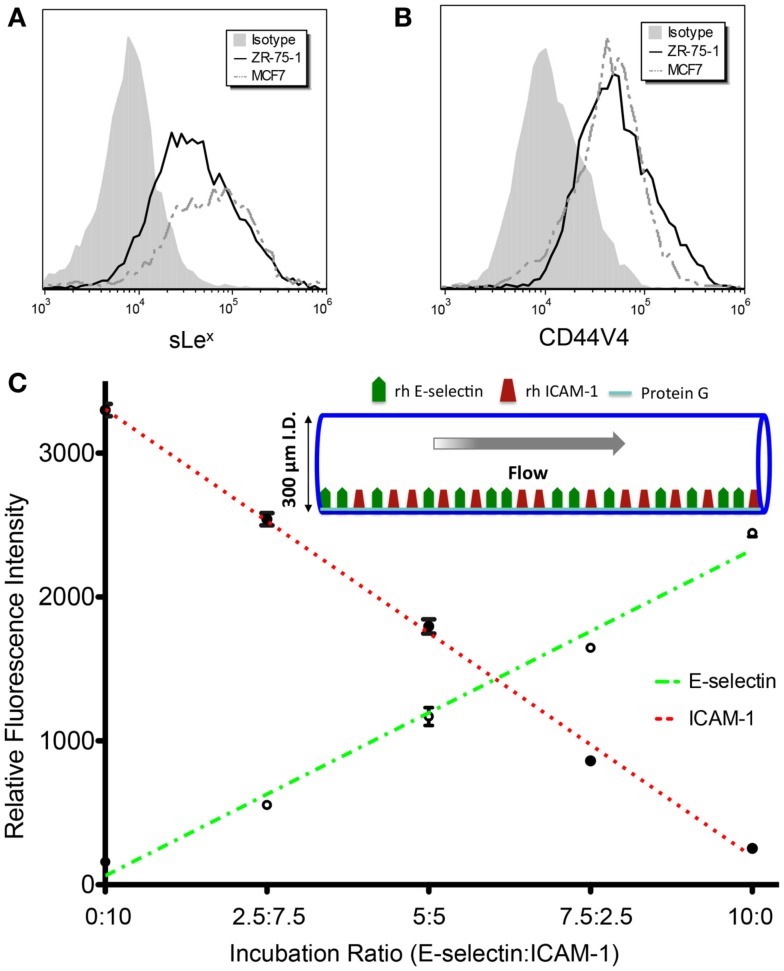
**(A,B)** Flow cytometry histogram overlays of ZR-75-1 and MCF7 cells labeled with anti-sLe^x^ (clone CSLEX) mAb and anti-CD44V4 mAb, respectively. **(C)** E-selectin and ICAM-1 fluorescence intensities as concentration ratios vary during surface preparation. The *r*^2^ values for E-selectin and ICAM-1 fluorescence intensity trend lines were found to be 0.986 and 0.995, respectively.

The MFI ratios of sample over isotype control for the E-selectin binding moiety sLe^x^ expression on ZR-75-1 and MCF7 were found to be 8.43 and 8.56, respectively, via flow cytometry (Figure 2A). CD44 variant 4 (CD44v4), among the multiple variants of common E-selectin ligand CD44, has been identified as a major E-selectin ligand for breast cancer cells (Zen et al., 2008). CD44v4 expression was measured on ZR-75-1 and MCF7 cells via flow cytometry and no significant difference was observed (Figure 2B).

### E-selectin and ICAM-1 combined surface

The initial layer of protein-G orients the adhesion molecules to maximally interact with cell surfaces as the cells are perfused through the tubes. As the E-selectin:ICAM-1 concentration ratios were increased, the fluorescence intensity of bound E-selectin in the microtube was found to linearly increase while ICAM-1 fluorescence linearly decreased (Figure 2C), verifying the desired protein concentrations on the microtube surfaces.

### MUC1 is involved in the cancer adhesion cascade in association with E-selectin and its ligands

**Figure 3:**
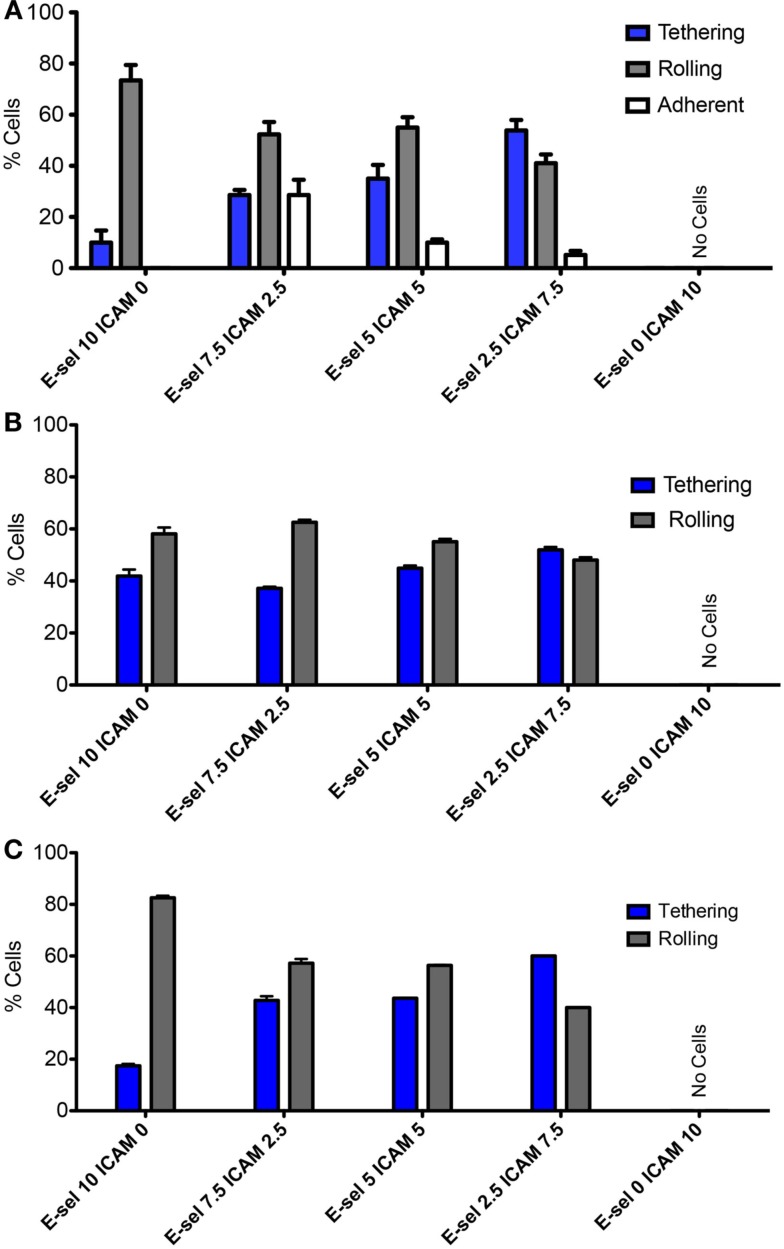
**Adhesion phenotypes on combined protein surface under physiological flow of (A) ZR-75-1, (B) MCF7, and (C) SM3 blocked ZR-75-1 cells**. Two-way ANOVA was used for statistical analysis and results from all conditions were found to be significantly different (*p* ≤ 0.001).

Figures 3A,B divide the cells that interact with the surface into three categories: tethering, rolling, and adherent. ZR-75-1 cells were found to roll quite consistently when E-selectin was present at any concentration on the surface where there was a slight decrease in the percent of rolling cells as the E-selectin concentration decreases (Figure 3A). Conversely, the percent of ZR-75-1 tethering cells increased as the E-selectin:ICAM-1 ratio decreased. Interestingly, adherent ZR-75-1 cells were observed only when both E-selectin and ICAM-1 exist on the surface and 7.5:2.5 was found to be the optimal ratio of E-selectin:ICAM-1 that yields the greatest number of adherent cells. However, as the E-selectin:ICAM-1 ratio decreased so did the percent of adherent cells. On the other hand, MCF7 rolling and tethering showed little sensitivity to varying the E-selectin:ICAM-1 ratios where MCF7 tethering cells only slightly increased as the ratio decreased, as shown in Figure 3B. Most notably, no adherent MCF7 cells were observed on the surface for any concentration ratio. For both ZR-75-1 and MCF7 cells, when only ICAM-1 coats the surface no cells interacted adhesively under flow. Anti-MUC1 mAb (clone SM3) was found to block the adhesive interactions of ZR-75-1 cells with the surface, leaving only the tethering and rolling populations (Figure 3C).

**Figure 4:**
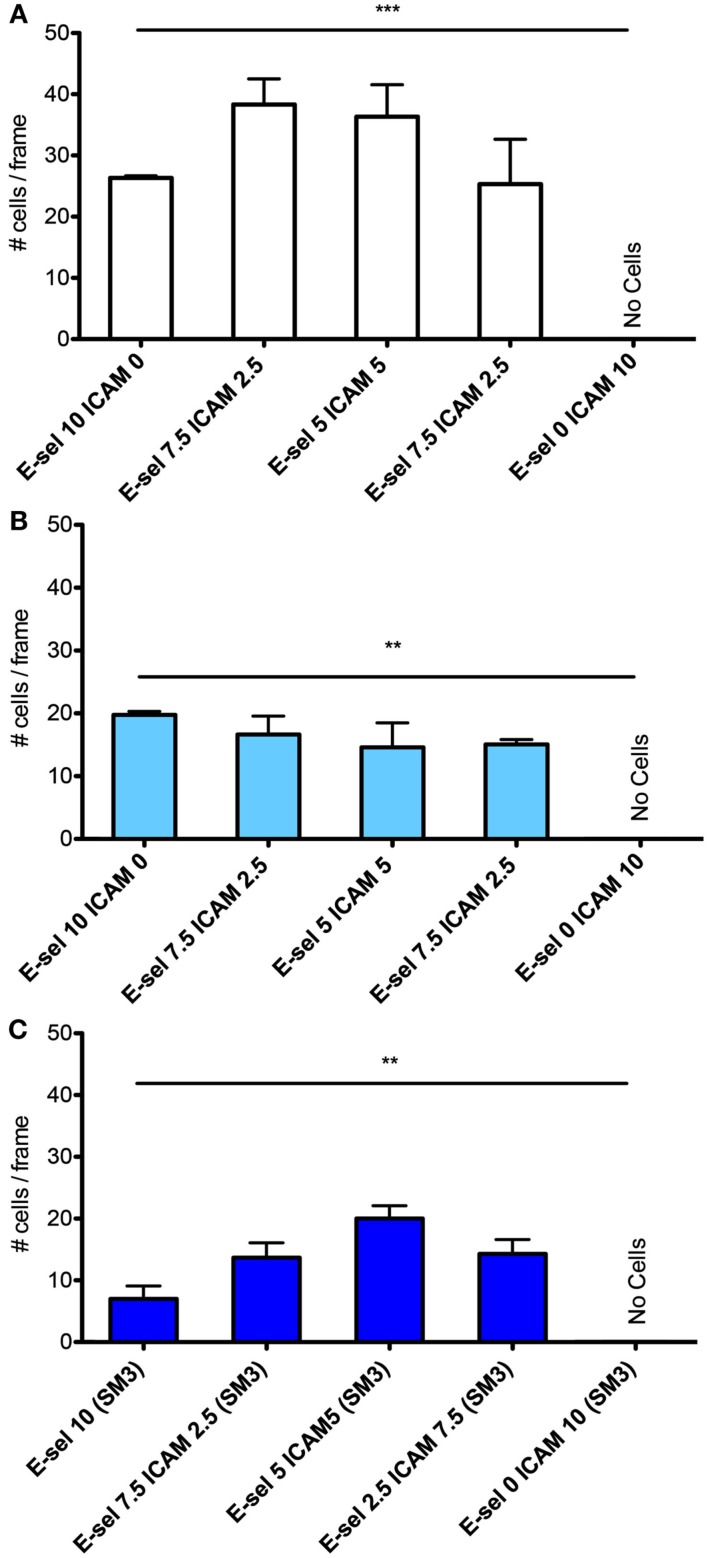
**Quantification of average number of cells captured on the surfaces with varying ratios of E-selectin and ICAM-1 for (A) ZR-75-1, (B) MCF7, and (C) SM3 blocked ZR-75-1 cells**. One-way ANOVA was used for statistical analysis. For all three experiments, results from all conditions were found to be significantly different with *p*-values of 0.0008, 0.0093, and 0.0016 for ZR-75-1, MCF7, and SM3 blocked ZR-75-1, respectively. **p ≤ 0.01; ***p ≤ 0.001.

**Figure 5:**
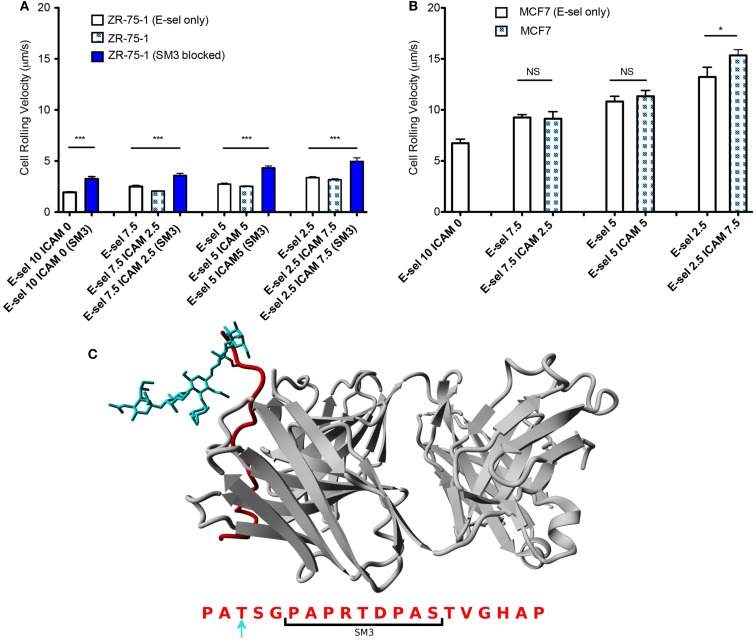
**(A,B)** Rolling velocities of ZR-75-1 (untreated and blocked) and MCF7 cells on combined and E-selectin surfaces with varying incubation concentrations under flow, respectively. **(C)** Equilibrated structure of SM3 (gray) bound to the core epitope of one repeat unit of MUC1 (red) singly O-glycosylated with sLe^x^ (blue) on a threonine residue. Below is the amino acid sequence of a MUC1 repeat unit where the bracket indicates the interaction site of SM3 and the blue arrow indicates the chosen O-glycosylated amino acid. Student’s *t*-test and ANOVA were used for statistical analysis. *p ≤ 0.05; ***p ≤ 0.001; NS, non-significant.

The cell flux for each cell type (Figures 4A–C) shows little sensitivity to the combined surface concentration ratios. However, overall the ZR-75-1 cell flux is much greater than both MCF7 and SM3 blocked ZR-75-1 cell fluxes, roughly by a factor of 2. Comparing ZR-75-1 to MCF7 and SM3 blocked ZR-75-1 cells, the cell fluxes inversely correlate with the cell rolling velocities, as shown in Figures 5A,B, where the ZR-75-1 cell rolling velocities were found to be slower than MCF7 cell rolling velocities. For example, for surfaces coated with only E-selectin, MCF7 cells rolled at 6.74 ± 0.40 μm/s whereas ZR-75-1 cells rolled at 1.93 ± 0.06 μm/s. SM3 blockade was found to cause an increase in rolling velocity to approximately 4 μm/s, significantly faster than untreated cells. A structure of SM3 bound to the underglycosylated core epitope of MUC1 is depicted in Figure 5C, where SM3 not only blocks ICAM-1 interactions, but is sufficiently bulky compared to sLe^x^ to inhibit some amount of E-selectin interactions as well. Unlike cell flux, both ZR-75-1 (untreated and blocked) and MCF7 cell rolling velocities were sensitive to the E-selectin concentration where cell rolling velocities increased as the E-selectin concentration decreased (Figures 5A,B). Furthermore, the MCF7 cell rolling velocity at the lowest E-selectin concentration was double that of the cell rolling velocity at the highest E-selectin concentration which shows a greater sensitivity to E-selectin compared to ZR-75-1 cells.

## Discussion

The detection and enumeration of CTCs holds great potential in breast, colorectal, and prostate cancer prognosis, yet the basic biophysics of how these CTCs interact with the endothelium is not fully understood. Similar to leukocyte recruitment to the endothelium, CTC tethering and rolling on the blood vessel wall under hydrodynamic shear stress are also mediated by the selectin family of adhesion molecules (Geng et al., 2012). After stable rolling on the endothelium, leukocytes can firmly adhere to the inflamed endothelium via leukocyte beta-2 integrin (Mac-1, LFA-1): ICAM-1 binding (Diamond et al., 1990; Ding et al., 1999; King, 2006). Similarly for epithelial-type CTCs, tumor associated MUC1 may play the role of beta-2 integrins on leukocytes by binding ICAM-1 to enable firm adhesion and initiate subsequent events in the metastatic cascade (Rahn et al., 2005).

Tumor associated MUC1 on breast cancer cells was first identified as a novel adhesion ligand for endothelial ICAM-1 by Regimbald et al. (1996) via static adhesion assays between MCF7 cells and stimulated HUVEC cells as well as immobilized recombinant ICAM-1. MCF7 cells do not express common ICAM-1 ligands, such as LFA-1, Mac-1, or CD43. However under static conditions, MUC1 was found to interact with ICAM-1, which could mediate firm adhesion of CTCs to the inflamed endothelium. In contrast, we characterized the adhesive role of tumor associated MUC1 under hydrodynamic shear stress by perfusing both ZR-75-1 (which overexpresses MUC1) and MCF7 cells through functionalized microtubes, more representative of the blood vessel microenvironment. Furthermore, the inflamed endothelium was simulated by immobilizing both E-selectin and ICAM-1 with varying ratios on the microtube surface, creating a more physiologically relevant and controllable environment to study the adhesion events of circulating ZR-75-1 and MCF7 cells under flow.

ZR-75-1 cells show a much greater expression of underglycosylated MUC1 compared to MCF7 cells, which significantly affects their adhesion behavior when perfused through the combined surface microtubes. Interestingly, although ZR-75-1 and MCF7 cells have similar expression levels of sLe^x^, one of the E-selectin binding moieties, ZR-75-1 cells roll on the combined protein surface at a significantly slower rolling velocity, indicating that ZR-75-1 cells establish stronger interactions with E-selectin. Recall that underglycosylated forms of MUC1 also contain shortened oligosaccharides where sLe^x^ is one of the most common carbohydrates of aberrant MUC1 (Burdick et al., 1997). Therefore MUC1, when appropriately decorated with sLe^x^ in its underglycosylated form, is expected to extend further from the cell surface compared to other selectin ligands due to its size and is perhaps more able to interact with E-selectin to efficiently mediate tethering and rolling events. The greater rolling velocities of ZR-75-1 cells blocked with SM3 also suggests MUC1 is an important E-selectin ligand because SM3 could inhibit some amount of E-selectin:MUC1 interactions due to the size of SM3 compared to sLe^x^ (Figure 5C). As a result of the slower rolling velocity and greater MUC1 expression, only ZR-75-1 cells firmly adhered to the combined surface, where firm adhesion is facilitated by MUC1:ICAM-1 interactions. In our study, the observation of firmly adhered cells to the combined surface under shear stress is consistent with the metastatic potential of the ZR-75-1 cell line (highly metastatic) and the MCF7 cell line (weakly metastatic).

**Figure 6:**
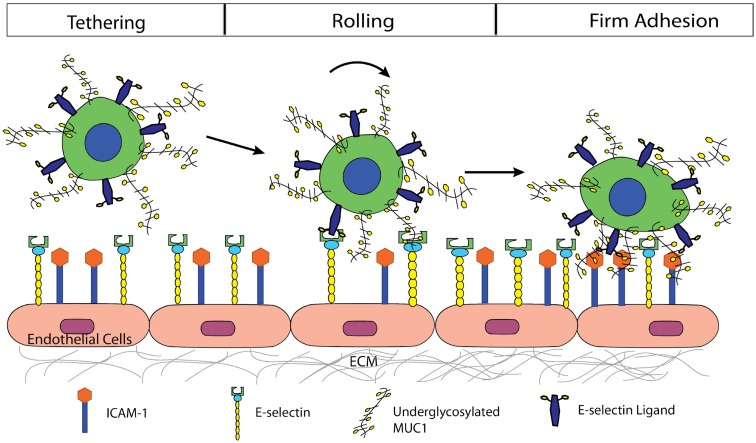
**Proposed mechanism by which MUC1 can act as a ligand for E-selectin, initiating tethering, and rolling events of CTCs on the endothelium, and subsequently serve as an ICAM-1 ligand, mediating firm adhesion of CTCs**.

In conclusion, we propose a mechanism by which MUC1 can act as a ligand for E-selectin, initiating tethering and rolling events of CTCs on the endothelium, and subsequently serve as an ICAM-1 ligand, mediating firm adhesion of CTCs (Figure 6). The synergistic effect of MUC1:E-selectin and MUC1:ICAM-1 may play an important role in breast cancer metastasis through the bloodstream where underglycosylated MUC1 can significantly slow the rolling velocity of CTCs thereby allowing for more frequent occurrence of firm adhesion events and subsequent extravasation. In summary, our results provide new insights into the roles of MUC1 in the metastatic adhesion cascade and suggests future examination into clinical aspects where the underglycosylated form of MUC1 can be targeted since aberrantly underglycosylated MUC1 expression is highly correlated to poor prognosis in breast and colon cancer patients.

## Conflict of Interest Statement

The authors declare that the research was conducted in the absence of any commercial or financial relationships that could be construed as a potential conflict of interest.

## Footnotes

^1^http://rsbweb.nih.gov/ij/

^2^http://yasara.org
